# B3 Superfamily in Cucumber (*Cucumis sativus* L.): Identification, Evolution, Expression Patterns, and Function in Glandular Trichome Development

**DOI:** 10.3390/ijms26094031

**Published:** 2025-04-24

**Authors:** Mingming Dong, Lei Sun, Wujun Wang, Yaru Wang, Li Shan, Xingwang Liu, Huazhong Ren

**Affiliations:** 1 Sanya Institute of China Agricultural University, Sanya 572019, China; 2 Frontier Technology Research Institute of China Agricultural University in Shenzhen, Shenzhen 518119, China; 3 Department of Vegetable Science, College of Horticulture, China Agricultural University, Beijing 100193, China

**Keywords:** B3, cucumber, transcription factor, VIGS

## Abstract

The B3 transcription factor superfamily, crucial for plant growth and stress adaptation, remains poorly characterized in cucumber (*Cucumis sativus*), a globally important vegetable crop. Here, we conducted the first genome-wide identification of 52 B3 superfamily genes in cucumber, classifying them into LAV, ARF, RAV, and REM subfamilies through integrated phylogenetic and structural analyses. These genes exhibited conserved B3 domains with lineage-specific motif architectures and diverse exon–intron organizations, particularly within the structurally divergent REM subfamily. Collinearity analysis revealed segmental duplication as a key driver of family expansion, notably between syntenic REM clusters on chromosomes 2 (*CsREM5-7*) and 6 (*CsREM18-20*). Promoter *cis*-element profiling identified enrichment in hormone-responsive and stress adaptation motifs, suggesting functional diversification in signaling pathways. Furthermore, tissue-specific expression divergence was observed across 10 organs, with ARF members displaying broad regulatory roles and REM genes showing apical meristem enrichment. Strikingly, *CsRAV8* exhibited glandular trichome-specific expression, a novel finding, given *Arabidopsis* RAVs’ lack of trichome-related functions. Spatial validation via in situ hybridization localized *CsRAV8* transcripts to trichome glandular head cells. Functional investigation using virus-induced gene silencing (VIGS) demonstrated that *CsRAV8* suppression caused significant glandular trichome shriveling, implicating its role in maintaining glandular cavity integrity. This study provides the first comprehensive genomic inventory of B3 transcription factors in cucumber, providing evolutionary insights and functional frameworks for future functional genomics studies.

## 1. Introduction

The B3 superfamily constitutes a plant-exclusive transcription factor family characterized by the presence of at least one conserved B3 DNA-binding domain—a ~110-amino acid motif that mediates sequence-specific recognition of *cis*-regulatory elements in target promoters [1,2]. Ubiquitously distributed across the plant kingdom, this superfamily is phylogenetically categorized into four major subfamilies based on domain architecture and functional divergence: LAV (LEAFY COTYLEDON2 [LEC2]–ABSCISIC ACID INSENSITIVE3 [ABI3]–Viviparous ABI3 Like [VAL]) [3,4], ARF (Auxin Response Factor) [5], RAV (Related to ABI3 and VP1) [6], and REM (Reproductive Meristem) [7].

In *Arabidopsis thaliana*, 118 genes have been identified as B3 transcription factors [1]. Among those genes, the function of LAV, ARF and RAV subfamily genes has been well understood. The LAV family diverges into two functionally distinct clades: the LEC2–ABI3 subgroup, which transcriptionally activates seed maturation and embryonic identity through direct DNA binding, and the VAL subgroup, which represses embryonic programs and flowering transitions via epigenetic silencing [8,9,10]. ARF proteins often have another MR (middle region) and PB1 (Phox and Bem 1), except for the B3 DNA-binding domain [11,12]. As the central regulators of auxin signaling, ARFs orchestrate diverse developmental processes and stress adaptation, including organ polarity, floral abscission, and salt stress [13,14,15]. Some RAV family members uniquely integrate dual DNA-binding domains (B3 and AP2), enabling recognition of bipartite motifs to suppress precocious flowering until developmental competence is attained [16,17,18]. In contrast, NGA genes—an RAV subgroup lacking the AP2 domain—regulate floral architecture and leaf morphogenesis, with *Arabidopsis* mutants exhibiting pleiotropic defects such as malformed floral organs and aberrant leaf shapes [19,20,21]. The REM family typically harbors one or more B3 domains. Unlike the other well-understood subfamilies, few genes have been functionally analyzed [22,23]. One notable known REM gene is *VERNALIZATION1* (*VRN1*), which stabilizes epigenetic memory of cold exposure to maintain vernalization responses [24,25]. In conclusion, the B3 transcription factors are pivotal regulators of plant growth and stress adaptation.

As a key member of the Cucurbitaceae family, cucumber (*Cucumis sativus* L.) holds global agricultural significance as a major vegetable crop cultivated. Notably, the commercial value of cucumber fruits is critically dependent on their visual quality. The presence of bloom—composed of glandular trichomes (GTs) and their fragmented residues—represents a commercially detrimental trait that diminishes market appeal [26]. Therefore, elucidating the mechanisms of glandular trichome development could empower breeders to control bloom formation. Unlike the unicellular non-glandular trichomes in the model plant *Arabidopsis*, cucumber trichomes exhibit a distinct multicellular architecture, yet their developmental regulation remains largely unexplored. While a limited number of transcription factors have been implicated in the early development of multicellular trichomes, the regulatory mechanisms specifically governing glandular trichome (GT) morphogenesis within the Cucurbitaceae family remain poorly characterized. Notably, no studies to date have established a functional link between B3 transcription factors and GT morphogenesis in cucumber or related species.

Systematic identification of B3 genes has been completed in model plants such as *Arabidopsis* [1] and certain types of crops, including castor bean (*Ricinus communis*) [27], *Medicago truncatula* [28], soybean (*Glycine max*) [29], pineapple (*Ananas comosus* L.) [30], and pepper (*C. annuum*) [31]. In this study, we identified 52 B3 superfamily transcription factors in the cucumber genome, classifying them into four subfamilies (LAV, VAL, ARF, and REM) through integrated phylogenetic, structural, and synteny analyses. Notably, *CsRAV8* exhibited glandular trichome-specific expression, and preliminary functional validation via virus-induced gene silencing (VIGS) implicated its role in glandular trichome development. Our findings establish the first comprehensive genomic identification for cucumber B3 transcription factors, laying a foundation for future functional studies to unravel their roles in cucurbit development and stress resilience.

## 2. Results

### 2.1. Genome-Wide Identification and Phylogenetic Analysis of B3 Superfamily Genes in Cucumber

**Figure 1:**
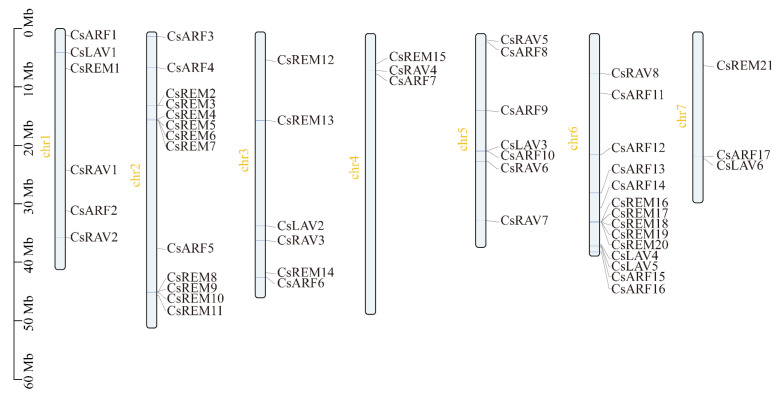
Chromosomal position of cucumber B3 genes. Gene names are annotated to the right of their respective loci, while the vertical scale on the left quantifies chromosomal length (Mb).

A total of 52 B3 genes, including 6 LAV, 8 RAV, 17 ARF, and 21 REM members, were identified in cucumber through integrated BlastP, Hidden Markov Model (HMM) profiling, conserved domain database (CDD) analysis, and Simple Modular Architecture Research Tool (SMART) screening (Table S1). These genes were systematically named based on their chromosomal localization and respective subfamily classification (Figure 1). The coding sequences ranged from 555 to 3324 bp in length, translating to proteins of 184–1107 amino acids with predicted molecular weights of 20.89–125.46 kDa. Isoelectric points (pIs) varied between 4.57 and 9.81 across all identified B3 proteins (Table S1). In addition, subcellular localization predictions indicated predominant nuclear localization for most cucumber B3 proteins, with additional membrane-associated and chloroplast-targeted isoforms identified.

**Figure 2:**
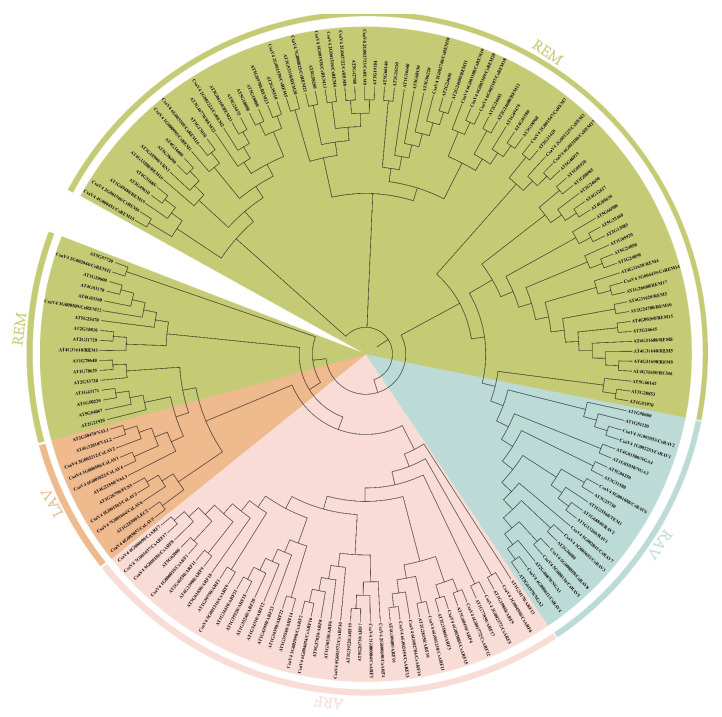
Phylogenetic analysis of cucumber B3 genes with previous reported *Arabidopsis* B3 genes. The corresponding subfamilies are color-coded for distinction.

To elucidate evolutionary relationships, we constructed a phylogenetic tree containing these 52 cucumber B3 genes and 118 *Arabidopsis* B3 superfamily homologs (Figure 2). The analysis revealed conserved clustering patterns. Cucumber LAV, RAV, and ARF subfamilies formed well-supported clades with their Arabidopsis counterparts. However, REM family members exhibited divergent phylogenetic relationships, suggesting substantial functional diversification within this gene family.

### 2.2. Gene Structural and Protein Motif Analysis

**Figure 3:**
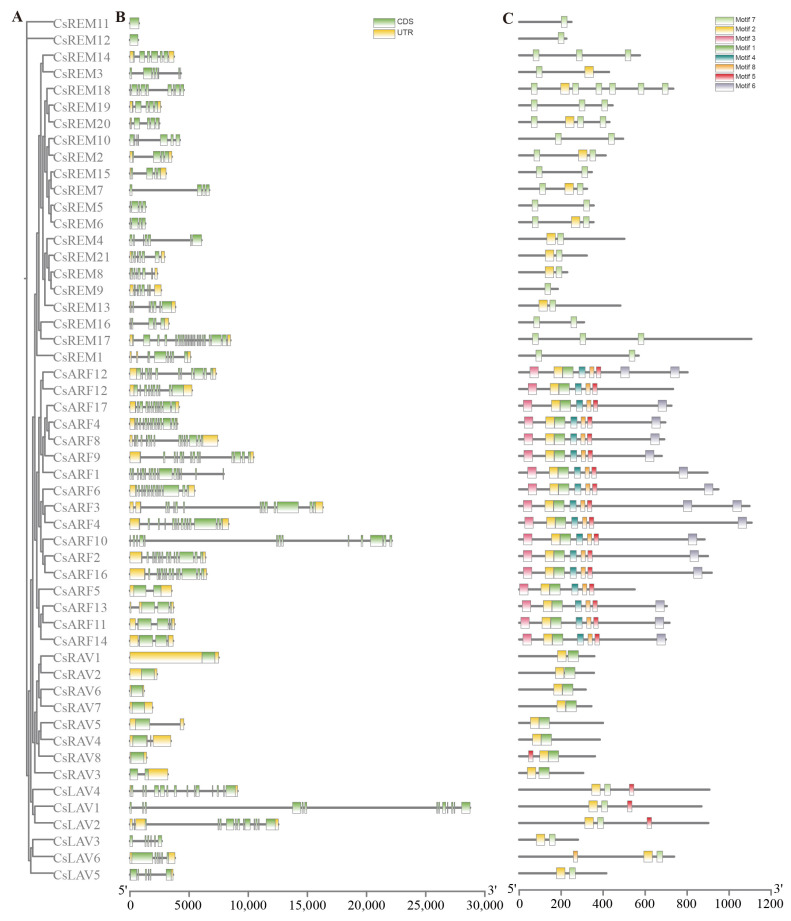
Gene structure and conserved motifs of cucumber B3 genes. (**A**) The phylogenetic tree illustrating the subfamily classification. (**B**) Gene structure of B3 genes are represented, with coding sequences (CDSs) in green boxes and untranslated regions (UTRs) in yellow boxes. (**C**) Eight conserved motifs are highlighted with different colors.

Comprehensive investigation of cucumber B3 gene features encompassed systematic examination of gene architecture and sequence characteristics. Phylogenetic reconstruction using MEGA 7 software generated an unrooted evolutionary tree that effectively distinguished B3 subfamily classifications (Figure 3A). As illustrated in Figure 3B, the 52 identified B3 genes exhibited considerable structural variation, with exon counts spanning 1–23 and corresponding intron numbers ranging 0–23. Notably, REM family members displayed particularly diverse intron–exon configurations, while RAV subfamily members predominantly exhibited single-exon structures.

Protein sequence conservation patterns were elucidated through MEME analysis, revealing critical insights into functional domain organization. All B3 proteins contained conserved B3 domains (represented by motifs 1, 2, and 7), with substantial motif conservation observed within each subfamily. ARF proteins demonstrated additional conservation of N-terminal motif 3 alongside characteristic auxin-response domains (motifs 4, 8, and 5). REM family members exhibited multiple B3 domain configurations, consistent with evolutionary patterns reported across diverse plant species (Figure 3C).

### 2.3. Collinearity Analysis of Cucumber B3 Genes

**Figure 4:**
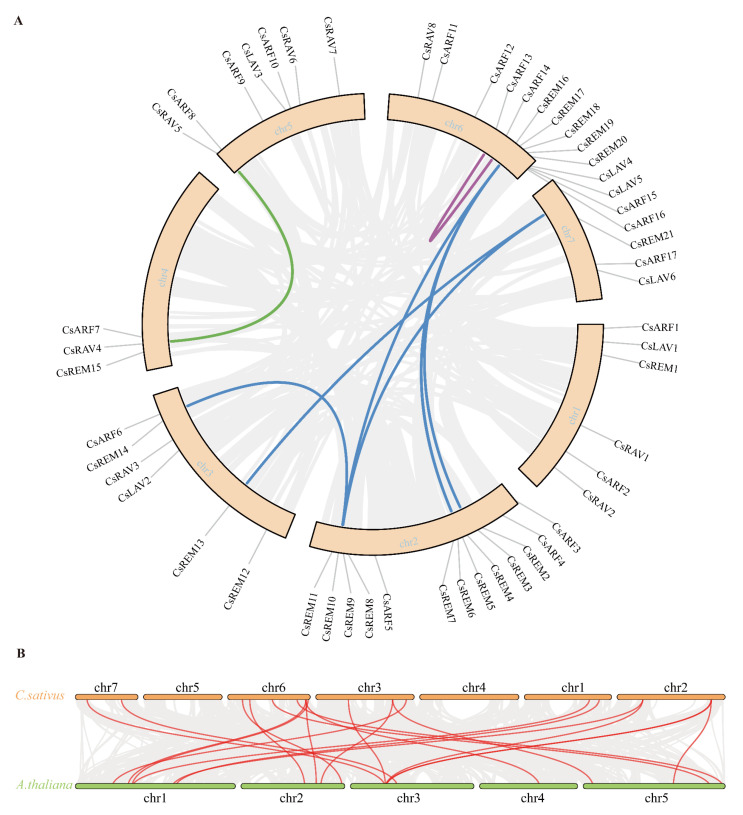
Collinearity analysis of B3 genes. (**A**) Collinear map of different B3 genes within cucumber. (**B**) Collinear map of B3 genes between cucumber and *Arabidopsis*. Gray lines show all gene duplications, while color lines show collinearity of B3 genes. The chromosome number and gene ID are illustrated.

Gene duplication serves as a critical mechanism for evolutionary innovation, facilitating the emergence of novel functional traits and driving the diversification of multigene families [32]. In the current investigation, we performed comparative syntenic analyses to elucidate genomic collinearity patterns among B3 transcription factor loci both intra-genomically within *Cucumis sativus* and inter-genomically between cucumber and *Arabidopsis*, employing the MCScanX computational framework (Figure 4). Ten collinear gene pairs were found within fifty-two cucumber B3 genes. Notably, a tightly linked gene cluster (*CsREM5*, *CsREM6*, *CsREM7*) on Chromosome 2 exhibited one-to-one correspondence with another cluster (*CsREM20*, *CsREM19*, *CsREM18*) on Chromosome 6, suggesting a large-scale segmental duplication event in this chromosomal region. Evolutionary analysis through the calculation of nonsynonymous (Ka) and synonymous (Ks) substitution rates revealed that all ten gene pairs displayed Ka/Ks ratios < 1, indicating their evolution under purifying selection. Particularly, the *CsREM5*–*CsREM20* pair showed a Ka/Ks ratio approaching 0, implying potential functional redundancy between these paralogs. To further investigate the evolutionary mechanisms of cucumber B3 genes, we analyzed collinear relationships between cucumber and *Arabidopsis* genomes, identifying 21 conserved orthologous gene pairs between cucumber and *Arabidopsis* (Figure 4).

### 2.4. Analysis of Cis-Acting Elements in Cucumber B3 Gene Promoters

**Figure 5:**
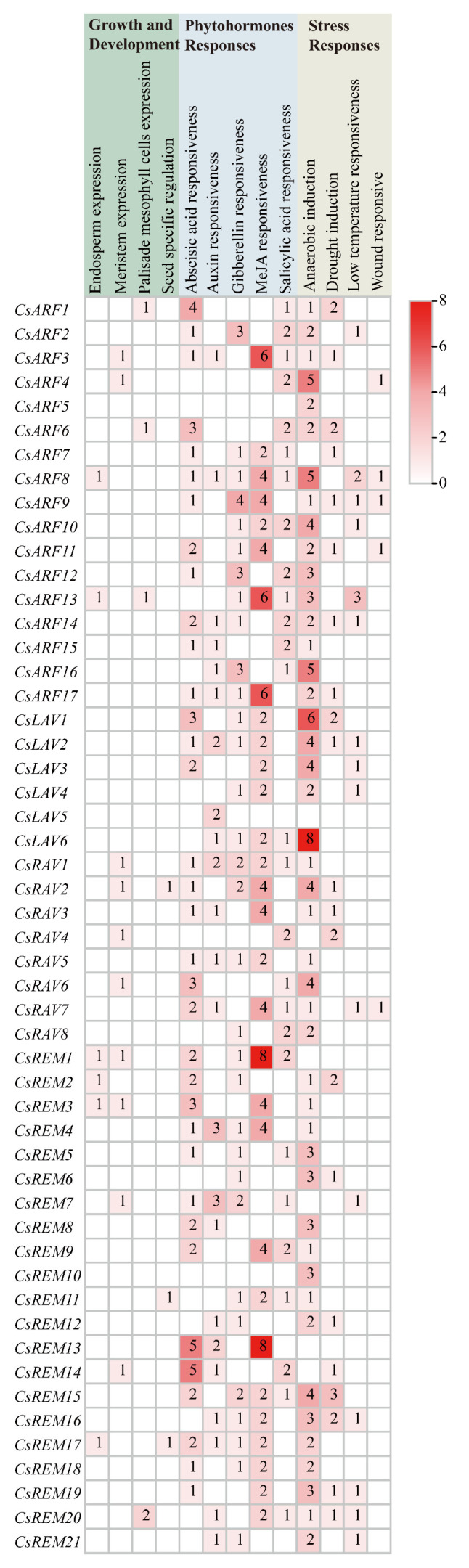
The *cis*-acting element analysis of cucumber B3 gene’s promoters. The numbers of *cis*-acting elements are shown in the heatmap.

To investigate the regulatory mechanisms of cucumber B3 genes, we performed *cis*-acting element analysis on promoter regions (defined as the 2000 bp upstream from the ATG start codon) of all 52 cucumber B3 genes using the Plant *Cis*-Acting Regulatory Element (Plant CARE) database. Thirteen distinct *cis*-acting regulatory elements were identified and categorized into three major functional classes: “Growth and Development”, “Phytohormone Responsiveness”, and “Stress Responses” (Figure 5). Notably, multiple phytohormone- and stress-responsive elements were prevalent across most genes, with *CsARF3*, *CsARF13*, *CsARF17*, *CsREM1*, and *CsREM13* exhibiting particularly high frequencies of methyl jasmonate (MeJA)-responsive elements. Tissue-specific regulatory elements were identified in several promoters, including meristem-specific elements in *CsARF3* and *CsARF4*, along with seed-specific elements in *CsRAV2*, *CsREM11*, and *CsREM17*. These spatial expression signatures suggest potential organ-specific transcriptional regulation patterns for corresponding genes. The comprehensive profiling of promoter architectures provides critical insights into the molecular basis of B3 gene regulation, particularly highlighting their involvement in hormonal signaling pathways and environmental adaptation mechanisms.

### 2.5. Expression Pattern of Cucumber B3 Genes

The rapid advancement of high-throughput sequencing technologies has enabled widespread generation of high-quality transcriptomic datasets across various cucumber organs. Leveraging these publicly accessible resources [33,34], we performed comprehensive transcriptional profiling of 52 identified B3 superfamily genes in cucumber, systematically characterizing their expression patterns across 10 distinct tissue types: root, stem, leaf, tendril, female flower, male flower, ovary, fruit flesh, glandular trichomes, and non-glandular trichomes.

**Figure 6:**
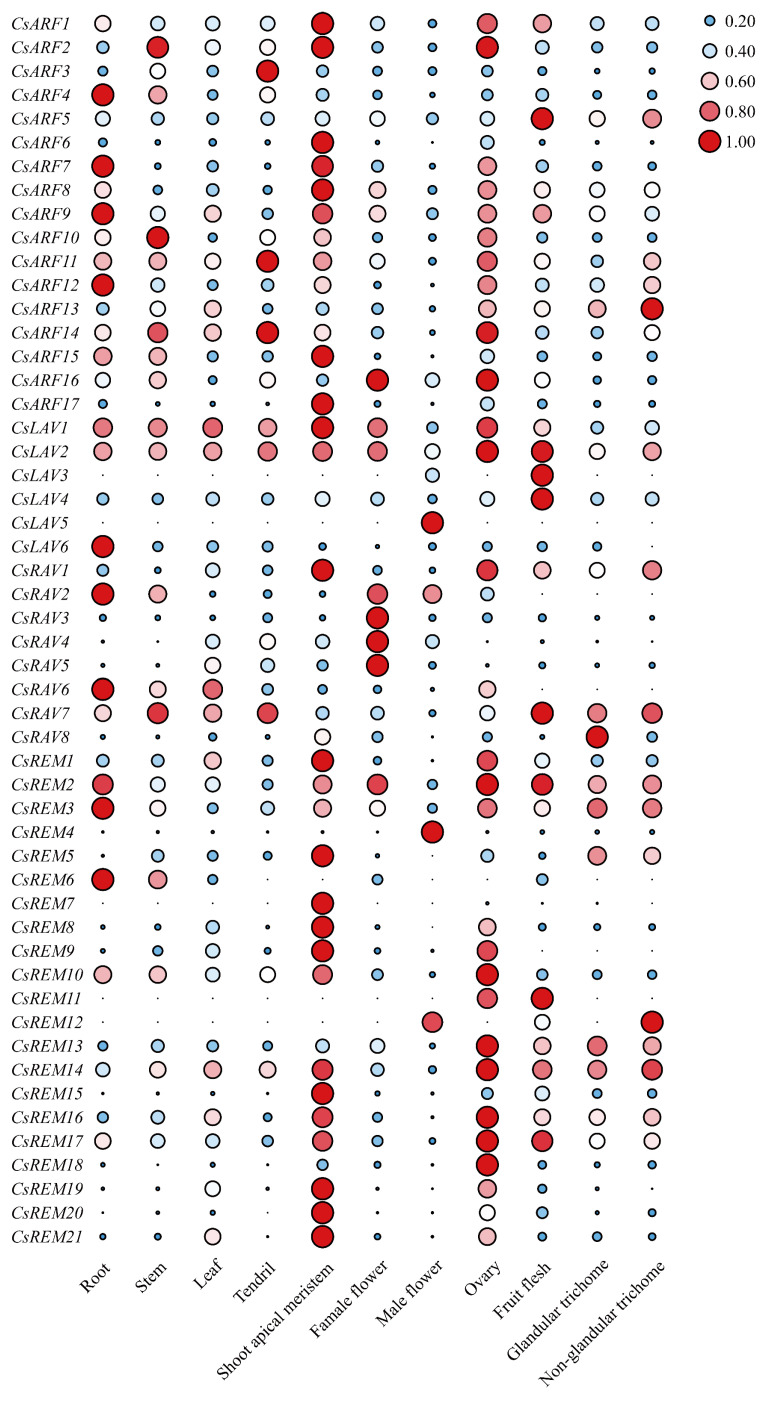
The expression patterns of cucumber B3 genes in different tissues of cucumber. For each gene, the FPKM-normalized value (by dividing gene expression in different tissues with the maximum observed FPKM) is shown. Expression gradient: blue (low) to red (high) (see the color bar at the right of the figure).

As illustrated in Figure 6, B3 members exhibited marked tissue-specific expression divergence, particularly within the ARF subfamily. Almost each ARF gene demonstrated unique spatial expression patterns, consistent with their established roles as auxin-responsive transcription factors governing morphogenesis in multiple plant tissues. In contrast to the diverse expression patterns observed among ARF members, the majority of REM family genes exhibit preferential expression in the shoot apical meristem (SAM), suggesting their potential involvement in early organogenesis. Only a subset of REM members demonstrated organ-specific enrichment: *CsREM4* displayed predominant expression in male flowers, *CsREM6* in roots, and *CsREM18* in ovaries. This spatial expression specialization implies functional diversification within the REM family, with distinct members likely contributing to developmental processes in specific tissues.

The LAV subfamily presented divergent expression patterns: *CsLAV1* and *CsLAV2* maintained constitutive expression across all tissues, whereas *CsLAV3*-*CsLAV6* exhibited discrete localization in ovaries (*CsLAV3-4*), male flowers (*CsLAV5*), and roots (*CsLAV6*). Notably, *CsLAV6* showed exclusive male flower expression with undetectable levels in other organs. RAV members (*CsRAV2/3/5/6*) demonstrated female flower-specific accumulation, potentially regulating gynoecium development. Strikingly, *CsRAV8* exhibited glandular trichome-specific expression—a novel finding given the absence of both glandular trichomes and documented RAVs’ involvement in trichome biology in *Arabidopsis*.

### 2.6. Preliminary Functional Validation of CsRAV8

The transcriptional profiling data demonstrated specific accumulation of *CsRAV8* transcripts in glandular trichomes, a noteworthy discovery as no previous studies have documented RAV family genes’ involvement in trichome morphogenesis. The specific expression pattern of *CsRAV8* prompted our investigation into its function.

**Figure 7:**
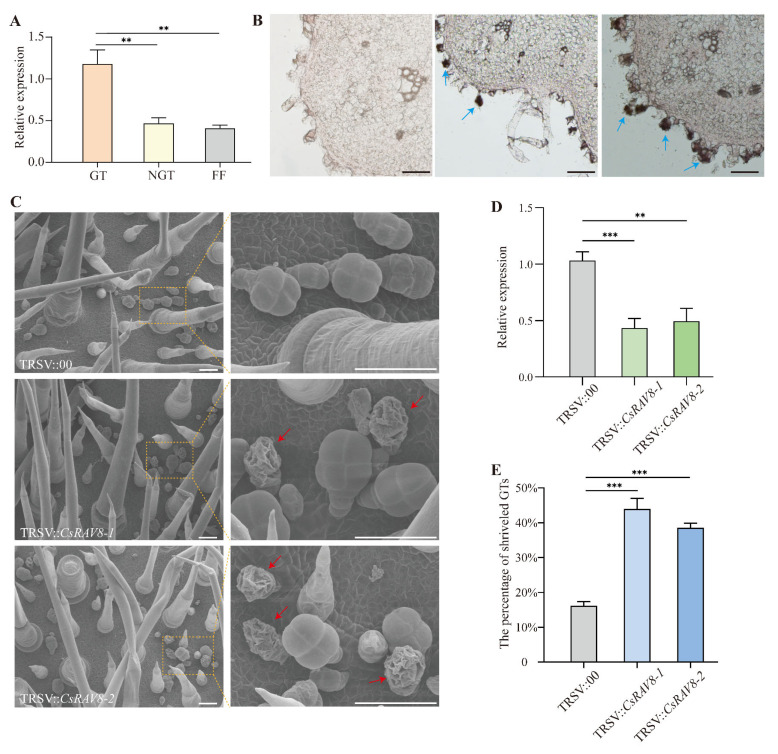
Expression profiling and functional validation of *CsRAV8*. (**A**) Expression analysis of *CsRAV8* in cucumber glandular trichomes (GTs), non-glandular trichomes (NGTs) and fruit flesh (FF). (**B**) In situ hybridization performed in young cucumber ovary. The blue arrows indicate staining-positive GTs. Bar = 50 μm. (**C**) Scanning electron microscopy (SEM) images of CsRAV8-silenced and control plants; red arrows indicate shriveled glandular trichomes (GTs). Bar = 50 μm. (**D**) Expression analysis of *CsRAV8* in respective VIGS-silenced plants. (**E**) Quantification of the proportion of shriveled GTs in respective VIGS-silenced plants. ** *p* < 0.01; *** *p* < 0.001, Student’s *t* test.

Primary validation through quantitative reverse transcription PCR (qRT-PCR) analysis confirmed significantly elevated *CsRAV8* expression in glandular trichomes compared to non-glandular trichomes and fruit flesh (Figure 7A), confirming transcriptional profiling results. Spatial resolution via in situ hybridization revealed intense hybridization signals exclusively localized in secretory head cells of glandular trichomes, with minimal background signals in negative controls (Figure 7B). The convergent evidence from quantitative and spatial expression analyses conclusively establishes *CsRAV8′*s glandular head-specific expression signature.

Functional characterization using virus-induced gene silencing (VIGS) demonstrated a marked phenotypic alteration, with silenced plants exhibiting 44.0% and 38.5% shriveled glandular trichomes compared to 16.1% in wild-type controls (Figure 7C–E). Considering the established association between trichome collapse and senescence dynamics [35], these findings suggest *CsRAV8′*s potential regulatory role in preserving secretory cavity integrity. Collectively, our data identify *CsRAV8* as a glandular trichome-specific regulator implicated in maintaining secretory compartment homeostasis.

## 3. Discussion

The B3 superfamily of transcription factors plays pivotal roles in diverse aspects of plant development and stress adaptation. Systematic investigation of these genes provides a foundational genomic resource for understanding their roles in crop development and stress adaptation. Genome-wide studies have identified B3 members across diverse plant lineages, while functional characterization in *Arabidopsis thaliana*—which hosts 118 annotated B3 genes—has established well-defined templates for key subgroups such as the LAV family, RAV clade, and auxin-responsive ARF proteins. Leveraging the newly released high-quality genome of cucumber (*Cucumis sativus*), this study identified 52 B3 genes, each encoding the conserved B3 DNA-binding domain. Notably, the cucumber B3 family displays significant contraction compared to *Arabidopsis*, yet the LAV subfamily retains conserved gene numbers, a trend also observed in castor bean (*Ricinus communis*) [27] and pineapple (*Ananas comosus*) [30], suggesting evolutionary constraints imposed by its conserved regulatory roles. In contrast, REM subfamily members exhibit marked numerical divergence across species, and their functional roles remain poorly characterized, highlighting a critical knowledge gap and a promising avenue for future research into plant transcriptional regulation.

The evolutionary expansion of plant gene families is predominantly mediated through tandem duplication, segmental duplication, and transposition events, with segmental duplication contributing more substantially than tandem duplication across most species [31,36]. In this study, collinearity analysis of cucumber B3 genes identified 10 syntenic gene pairs, including a syntenic cluster on Chromosome 2 (*CsREM5*, *CsREM6*, and *CsREM7*) exhibiting precise positional conservation with a homologous cluster (*CsREM18-20*) on Chromosome 6, strongly supporting ancestral segmental duplication events between these regions. These findings refine models of B3 family evolution in Cucurbitaceae. Comparative genomic analysis further revealed 21 orthologous gene pairs between cucumber and *Arabidopsis*, where evolutionary conservation typically predicts functional equivalence. However, expression profiling uncovered unexpected functional divergence among these syntenic orthologs: while *CsARF2* displays predominant expression in meristems and ovaries, its Arabidopsis counterpart *AtARF6* regulates stamen elongation [37]. Similarly, *AtARF10*, essential for root cap cell differentiation in Arabidopsis [38], contrasts with its cucumber ortholog *CsARF11*, which exhibits preferential tendril-specific expression. This functional decoupling suggests highlighting possibly lineage-specific neofunctionalization of conserved gene lineages.

Cucumber trichomes display remarkable morphological complexity distinct from the simple unicellular, non-glandular structures of *Arabidopsis*, comprising eight multicellular types categorized as glandular or non-glandular [39]. Notably, glandular trichomes in cucumber serve dual roles as defense structures and key determinants of fruit quality through specialized metabolite secretion [26]. To investigate potential associations between B3 transcription factors and multicellular trichome development, we analyzed their expression across standard plant tissues and specifically within glandular/non-glandular trichomes. Transcriptomic profiling identified a subset of B3 genes—including *CsARF13*, *CsRAV7*, *CsRAV8*, *CsREM2*, *CsREM3*, *CsREM12*, *CsREM13*, and *CsREM14*—with elevated expression in trichomes. Strikingly, *CsRAV8* exhibited exclusive glandular trichome-specific expression, virtually undetectable in other tissues. This finding carries evolutionary significance, as the model plant *Arabidopsis* lacks glandular trichomes, suggesting RAV subfamily neofunctionalization in cucumber. qRT-PCR and in situ hybridization confirmed *CsRAV8′*s spatial specificity, while virus-induced gene silencing (VIGS) revealed its critical role in maintaining secretory cavity integrity within glandular trichomes. These results establish novel regulatory roles for B3 transcription factors in cucumber trichome morphogenesis and identify a potential target for improving cucumber resistance through targeted trichome engineering.

## 4. Materials and Methods

### 4.1. Gene Identification and Chromosomal Locations

The identification of cucumber B3 superfamily genes were conducted through a multi-step approach. Initially, candidate genes were retrieved via BLAST_P alignment using *Arabidopsis* B3 protein sequences [1] as queries against the cucumber Chinese Long v4 genome database [33] (http://www.cucumberdb.com/#/home (accessed on 6 August 2024)), complemented by HMM-based profile searches with the conserved B3 domain (PF02362) as reference [40]. Subsequently, all putative sequences were subjected to structural validation through concurrent analysis in the NCBI Conserved Domain Database [41] (https://www.ncbi.nlm.nih.gov/Structure/bwrpsb/bwrpsb.cgi (accessed on 6 August 2024)) and Simple Modular Architecture Research Tool (SMART) [42] (http://smart.embl-heidelberg.de (accessed on 7 August 2024)) platform to verify complete B3 domain architecture. Molecular weights and isoelectric points were calculated using ProtParam tool on the ExPASy server [43] (https://web.expasy.org/protparam (accessed on 7 August 2024)), while WoLF PSORT (http://www.genscript.com/psort/wolf_psort.html (accessed on 7 August 2024)) predicted subcellular localization probabilities through k-nearest neighbor classification. Chromosomal positions were determined using genome coordinate data from the Chinese Long v4 genome assembly, with physical mapping visualizations generated through TBtools (v2.210) genome annotation module [44].

### 4.2. Phylogenetic Analysis

The global alignment of *Arabidopsis* and cucumber B3 gene sequences was performed with ClustalW, followed by phylogenetic reconstruction through the neighbor-joining algorithm in MEGA 7. Parameters included 1000 bootstrap replicates, Poisson substitution model, and complete gap removal. The resultant evolutionary tree underwent topological refinement and graphical annotation using the iTOL platform (https://itol.embl.de (accessed on 15 August 2024)).

### 4.3. Gene Structure Analysis

The exon–intron architecture of cucumber B3 genes was constructed utilizing TBtools (v2.210) software, while conserved protein motifs were systematically identified through the MEME algorithm [45]. Subsequent graphical representations of these analytical outputs were produced and refined within the TBtools (v2.210) visualization platform [44].

### 4.4. Synteny Analysis

The identification of tandem and segmental duplication events in B3 genes was performed using the Multiple Collinearity Scan toolkit (MCscanX (v1.0.0)) [46], with corresponding collinearity maps subsequently generated through TBtools (v2.210). Evolutionary parameters including synonymous (Ks) and nonsynonymous (Ka) substitution rates were determined via computational analysis in DnaSP 5 [47].

### 4.5. Cis-Acting Element Analysis

The 2 kb promoter region upstream of the start codon of each gene was downloaded from cucumber Chinese Long V4 genome to analyze the possible *cis*-acting elements of B3 genes by the PlantCARE (http://bioinformatics.psb.ugent.be/webtools/plantcare/html/ (accessed on 16 August 2024)) online server [48].

### 4.6. Expression Profiling Analysis

Transcriptomic datasets encompassing root, stem, leaf, tendril, female flower, male flower, and ovary tissues of cucumber were obtained from Guan et al. (2024) [33], while glandular and non-glandular trichome expression profiles were derived from Feng et al. (2023) [34]. The normalized gene expression profiles were visualized using TBtools (v2.210) [41]. To achieve normalization, expression values in each tissue were scaled relative to the maximum expression level observed across all tissues (e.g., for *CsARF1* which demonstrated peak expression in the shoot apical meristem, tissue-specific expression values were divided by this maximal value to obtain normalized expression ratios). This normalization approach enables cross-tissue comparison by establishing a standardized reference point based on the tissue exhibiting maximum transcriptional activity for each gene.

### 4.7. RNA Extraction and Real-Time Quantitative PCR (RT-qPCR) Analysis

Fruits of the East Asian cucumber ecotype CCMC harvested at anthesis were dissected to isolate glandular trichomes, non-glandular trichomes, and fruit flesh tissues. Total RNA was extracted from these tissues using the Huayueyang RNA Isolation Kit (Huayueyang Biotechnology, Beijing, China) according to the manufacturer’s instructions. First-strand cDNA synthesis was subsequently performed using the PrimeScript RT Reagent Kit with gDNA Eraser (Takara Bio, Shiga, Japan). Quantitative reverse transcription PCR (RT-qPCR) analysis was carried out on an ABI 7500 Real-Time PCR System (Applied Biosystems, Waltham, MA, USA) using SYBR Premix Ex Taq II (Takara Bio, Shiga, Japan) in 96-well optical plates. Each experimental condition, consisting of different cDNA samples and specific primer pairs, was analyzed with triplicate biological replicates and three technical replicates. The cucumber *α-TUBULIN* gene was used as an endogenous control for normalization. The gene-specific primers used for qPCR are listed in Table S2.

### 4.8. In Situ Hybridization

Young fruits were fixed in 3.7% FAA (formalin-acetic acid-alcohol) solution. For in situ hybridization, SP6 and T7 RNA polymerases were employed to generate sense and antisense RNA probes, respectively. All procedures including tissue fixation, paraffin embedding, microtome sectioning, and hybridization followed the standardized protocol established in Zhang et al.’s work (2021) [49]. The *CsRAV8* probe consisted of 285 bp linear DNA fragments encompassing the entire coding sequence. Primer sequences used for probe synthesis are documented in Table S2.

### 4.9. VIGS Assay

To investigate the functional roles of *CsRAV8* in cucumber, a tobacco ringspot virus-derived virus-induced gene silencing (VIGS) system was employed [50]. Unique 300–500 bp coding sequence (CDS) fragments (primer sequences detailed in Table S2) were cloned into the SnaBI restriction site of pTRSV2 vector, which was subsequently introduced into Agrobacterium tumefaciens strain GV3101. Cucumber seeds with emerging primary roots (≈1 cm length) underwent vacuum-mediated co-infiltration for 5 min using a suspension containing both pTRSV1 and recombinant pTRSV2 constructs. The seeds were then placed on half MS solid medium with 100 μM acetosyringone for 5 d. After that, the seedlings were planted in half Hoagland solution for another 15 d. The adaxial surface of the first true leaf was used to count the ratio of shriveled glandular trichomes.

### 4.10. Scanning Electron Microscopy

The samples were initially fixed in 2.5% (*v/v*) glutaraldehyde at 4 °C for approximately 24 h, followed by three PBS (pH 7.2) rinses. Post-fixation was performed using 1% (*v/v*) osmium tetroxide. Subsequent dehydration involved sequential immersion in ethanol gradients (30%, 50%, 70%, 80%, 90%, and 100%) with three repetitions at each concentration. Critical point drying was executed using a Hitachi HCP-2 desiccator, followed by gold-palladium sputter coating with an EIKO IB-3 system. Morphological characterization was conducted through Hitachi S-4700 scanning electron microscopy at 10 kV accelerating voltage.

## 5. Conclusions

This study provides the first comprehensive genomic identification of the B3 superfamily transcription factors in cucumber. Fifty-two identified B3 genes are classified into LAV, RAV, ARF, and REM subfamilies, each exhibiting distinct phylogenetic clustering, gene architecture, and motif organization. Collinearity analyses uncovered segmental duplication events and strong purifying selection shaping the evolutionary trajectory of these genes, while *cis*-regulatory element profiling highlighted their responsiveness to phytohormones and environmental stimuli. Tissue-specific expression patterns further underscored functional divergence: ARF members displayed broad regulatory roles across developmental stages, whereas REM genes were enriched in meristematic tissues, and RAV/LAV subgroups exhibited organ-specific localization. The discovery of *CsRAV8* as a glandular trichome-specific regulator marks a pivotal advancement, with functional validation via VIGS implicating its role in maintaining secretory head integrity—a novel finding expanding the known functional scope of RAV family genes beyond *Arabidopsis* paradigms. In conclusion, this study provides the first comprehensive genomic resource for B3 transcription factors in cucumber, thereby facilitating functional genomics investigations into their roles in cucurbit development and stress adaptation mechanisms.

## Data Availability

The authors responsible for the distribution of materials integral to the findings presented in this article are Huazhong Ren (renhuazhong@cau.edu.cn) and Xingwang Liu (liuxw01@cau.edu.cn).

## Supplementary Materials

The following supporting information can be downloaded at https://www.mdpi.com/article/10.3390/ijms26094031/s1.

## References

[1] Swaminathan K., Peterson K., Jack T. (2008). The plant B3 superfamily. Trends Plant Sci..

[2] Peng F.Y., Weselake R.J. (2013). Genome-wide identification and analysis of the B3 superfamily of transcription factors in Brassicaceae and major crop plants. Theor. Appl. Genet..

[3] Tsukagoshi H., Saijo T., Shibata D., Morikami A., Nakamura K. (2005). Analysis of a sugar response mutant of *Arabidopsis* identified a novel B3 domain protein that functions as an active transcriptional repressor. Plant Physiol..

[4] Suzuki M., Kao C.Y., McCarty D.R. (1997). The conserved B3 domain of VIVIPAROUS1 has a cooperative DNA binding activity. Plant Cell.

[5] Ulmasov T., Murfett J., Hagen G., Guilfoyle T.J. (1997). Aux/IAA proteins repress expression of reporter genes containing natural and highly active synthetic auxin response elements. Plant Cell.

[6] Chandan R.K., Kumar R., Swain D.M., Ghosh S., Bhagat P.K., Patel S., Bagler G., Sinha A.K., Jha G. (2023). RAV1 family members function as transcriptional regulators and play a positive role in plant disease resistance. Plant J..

[7] Franco-Zorrilla J.M., Cubas P., Jarillo J.A., Fernández-Calvín B., Salinas J., Martínez-Zapater J.M. (2002). AtREM1, a member of a new family of B3 domain-containing genes, is preferentially expressed in reproductive meristems. Plant Physiol..

[8] Boulard C., Fatihi A., Lepiniec L., Dubreucq B. (2017). Regulation and evolution of the interaction of the seed B3 transcription factors with NF-Y subunits. Biochim. Biophys. Acta (BBA)-Gene Regul. Mech..

[9] Chen N., Wang H., Abdelmageed H., Veerappan V., Tadege M., Allen R.D. (2020). HSI2/VAL1 and HSL1/VAL2 function redundantly to repress DOG1 expression in *Arabidopsis* seeds and seedlings. New Phytol..

[10] Chen C., Gong X., Li Y., Li H., Zhang H., Liu L., Liang D., Yuan W. (2022). Interaction Analysis between the *Arabidopsis* Transcription Repressor VAL1 and Transcription Coregulators SIN3-LIKEs (SNLs). Int. J. Mol. Sci..

[11] Chandler J.W. (2016). Auxin response factors. Plant Cell Environ..

[12] Roosjen M., Paque S., Weijers D. (2018). Auxin Response Factors: Output control in auxin biology. J. Exp. Bot..

[13] Li Y., Han S., Qi Y. (2023). Advances in structure and function of auxin response factor in plants. J. Integr. Plant Biol..

[14] Pekker I., Alvarez J.P., Eshed Y. (2005). Auxin response factors mediate *Arabidopsis* organ asymmetry via modulation of KANADI activity. Plant Cell.

[15] Marzi D., Brunetti P., Saini S.S., Yadav G., Puglia G.D., Dello Ioio R. (2024). Role of transcriptional regulation in auxin-mediated response to abiotic stresses. Front. Genet..

[16] Matias-Hernandez L., Aguilar-Jaramillo A.E., Marin-Gonzalez E., Suarez-Lopez P., Pelaz S. (2014). RAV genes: Regulation of floral induction and beyond. Ann. Bot..

[17] Hu H., Tian S., Xie G., Liu R., Wang N., Li S., He Y., Du J. (2021). TEM1 combinatorially binds to *FLOWERING LOCUS T* and recruits a Polycomb factor to repress the floral transition in *Arabidopsis*. Proc. Natl. Acad. Sci. USA.

[18] Feng C.Z., Chen Y., Wang C., Kong Y.H., Wu W.H., Chen Y.F. (2014). *Arabidopsis* RAV1 transcription factor, phosphorylated by SnRK2 kinases, regulates the expression of *ABI3*, *ABI4*, and *ABI5* during seed germination and early seedling development. Plant J..

[19] Trigueros M., Navarrete-Gomez M., Sato S., Christensen S.K., Pelaz S., Weigel D., Yanofsky M.F., Ferrandiz C. (2009). The NGATHA genes direct style development in the *Arabidopsis* gynoecium. Plant Cell.

[20] Fourquin C., Ferrandiz C. (2014). The essential role of NGATHA genes in style and stigma specification is widely conserved across eudicots. New Phytol..

[21] Ballester P., Navarrete-Gomez M., Carbonero P., Onate-Sanchez L., Ferrandiz C. (2015). Leaf expansion in *Arabidopsis* is controlled by a TCP-NGA regulatory module likely conserved in distantly related species. Physiol. Plant.

[22] Mantegazza O., Gregis V., Mendes M.A., Morandini P., Alves-Ferreira M., Patreze C.M., Nardeli S.M., Kater M.M., Colombo L. (2014). Analysis of the *Arabidopsis* REM gene family predicts functions during flower development. Ann. Bot..

[23] Caselli F., Beretta V.M., Mantegazza O., Petrella R., Leo G., Guazzotti A., Herrera-Ubaldo H., de Folter S., Mendes M.A., Kater M.M. (2019). *REM34* and *REM35* Control Female and Male Gametophyte Development in *Arabidopsis thaliana*. Front. Plant Sci..

[24] Levy Y.Y., Mesnage S., Mylne J.S., Gendall A.R., Dean C. (2002). Multiple roles of *Arabidopsis* VRN1 in vernalization and flowering time control. Science.

[25] Zhou H.B., Song Z.H., Zhong S., Zuo L.Y., Qi Z., Qu L.J., Lai L.H. (2019). Mechanism of DNA-Induced Phase Separation for Transcriptional Repressor VRN1. Angew. Chem.-Int. Edit..

[26] Feng Z., Bartholomew E.S., Liu Z., Cui Y., Dong Y., Li S., Wu H., Ren H., Liu X. (2021). Glandular trichomes: New focus on horticultural crops. Hortic. Res..

[27] Wang W.-B., Ao T., Zhang Y.-Y., Wu D., Xu W., Han B., Liu A.-Z. (2022). Genome-wide analysis of the B3 transcription factors reveals that RcABI3/VP1 subfamily plays important roles in seed development and oil storage in castor bean (*Ricinus communis*). Plant Divers..

[28] Gao J., Ma G., Chen J., Gichovi B., Cao L., Liu Z., Chen L. (2024). The B3 gene family in Medicago truncatula: Genome-wide identification and the response to salt stress. Plant Physiol. Biochem..

[29] Ren C., Wang H., Zhou Z., Jia J., Zhang Q., Liang C., Li W., Zhang Y., Yu G. (2022). Genome-wide identification of the B3 gene family in soybean and the response to melatonin under cold stress. Front. Plant Sci..

[30] Ruan C.C., Chen Z., Hu F.C., Fan W., Wang X.H., Guo L.J., Fan H.Y., Luo Z.W., Zhang Z.L. (2021). Genome-wide characterization and expression profiling of B3 superfamily during ethylene-induced flowering in pineapple (*Ananas comosus* L.). BMC Genom..

[31] Park Y.S., Cho H.J., Kim S. (2024). Identification and expression analyses of B3 genes reveal lineage-specific evolution and potential roles of REM genes in pepper. BMC Plant Biol..

[32] Cannon S.B., Mitra A., Baumgarten A., Young N.D., May G. (2004). The roles of segmental and tandem gene duplication in the evolution of large gene families in *Arabidopsis thaliana*. BMC Plant Biol..

[33] Guan J., Miao H., Zhang Z., Dong S., Zhou Q., Liu X., Beckles D.M., Gu X., Huang S., Zhang S. (2024). A near-complete cucumber reference genome assembly and Cucumber-DB, a multi-omics database. Mol. Plant.

[34] Feng Z.X., Sun L., Dong M.M., Fan S.S., Shi K.X., Qu Y.X., Zhu L.Y., Shi J.F., Wang W.J., Liu Y.H. (2023). Novel players in organogenesis and flavonoid biosynthesis in cucumber glandular trichomes. Plant Physiol..

[35] Dong M., Xue S., Bartholomew E.S., Zhai X., Sun L., Xu S., Zhang Y., Yin S., Ma W., Chen S. (2022). Transcriptomic and functional analysis provides molecular insights into multicellular trichome development. Plant Physiol..

[36] Jiang S.Y., González J.M., Ramachandran S. (2013). Comparative Genomic and Transcriptomic Analysis of Tandemly and Segmentally Duplicated Genes in Rice. PLoS ONE.

[37] Zhou D., Song R.Q., Fang Y., Liu R., You C.J., Wang Y.J., Huang L. (2025). Global identification and regulatory network analysis reveal the significant roles of lncRNAs during anther and pollen development in *Arabidopsis*. Plant Cell Rep..

[38] Cai X.X., Zhang H., Mu C.Q., Chen Y.J., He C.Z., Liu M.Y., Laux T., Pi L.M. (2025). A mobile miR160-triggered transcriptional axis controls root stem cell niche maintenance and regeneration in *Arabidopsis*. Dev. Cell.

[39] Xue S., Dong M., Liu X., Xu S., Pang J., Zhang W., Weng Y., Ren H. (2019). Classification of fruit trichomes in cucumber and effects of plant hormones on type II fruit trichome development. Planta.

[40] Bateman A., Coin L., Durbin R., Finn R.D., Hollich V., Griffiths-Jones S., Khanna A., Marshall M., Moxon S., Sonnhammer E.L. (2004). The Pfam protein families database. Nucleic Acids Res..

[41] Lu S., Wang J., Chitsaz F., Derbyshire M.K., Geer R.C., Gonzales N.R., Gwadz M., Hurwitz D.I., Marchler G.H., Song J.S. (2020). CDD/SPARCLE: The conserved domain database in 2020. Nucleic Acids Res..

[42] Letunic I., Khedkar S., Bork P. (2021). SMART: Recent updates, new developments and status in 2020. Nucleic Acids Res..

[43] Gasteiger E., Gattiker A., Hoogland C., Ivanyi I., Appel R.D., Bairoch A. (2003). ExPASy: The proteomics server for in-depth protein knowledge and analysis. Nucleic Acids Res..

[44] Chen C.J., Wu Y., Li J.W., Wang X., Zeng Z.H., Xu J., Liu Y.L., Feng J.T., Chen H., He Y.H. (2023). TBtools-II: A “one for all, all for one” bioinformatics platform for biological big-data mining. Mol. Plant.

[45] Bailey T.L., Johnson J., Grant C.E., Noble W.S. (2015). The MEME Suite. Nucleic Acids Res..

[46] Wang Y., Li J., Paterson A.H. (2013). MCScanX-transposed: Detecting transposed gene duplications based on multiple colinearity scans. Bioinformatics.

[47] Librado P., Rozas J. (2009). DnaSP v5: A software for comprehensive analysis of DNA polymorphism data. Bioinformatics.

[48] Lescot M., Déhais P., Thijs G., Marchal K., Moreau Y., Van de Peer Y., Rouzé P., Rombauts S. (2002). PlantCARE, a database of plant *cis*-acting regulatory elements and a portal to tools for in silico analysis of promoter sequences. Nucleic Acids Res..

[49] Zhang Y., Shen J., Bartholomew E.S., Dong M., Chen S., Yin S., Zhai X., Feng Z., Ren H., Liu X. (2021). TINY BRANCHED HAIR functions in multicellular trichome development through an ethylene pathway in *Cucumis sativus* L. Plant J. Cell Mol. Biol..

[50] Fang L., Wei X.Y., Liu L.Z., Zhou L.X., Tian Y.P., Geng C., Li X.D. (2021). A tobacco ringspot virus-based vector system for gene and microRNA function studies in cucurbits. Plant Physiol..

